# Environmental Surveillance for Risk Assessment in the Context of a Phase 2 Clinical Trial of Type 2 Novel Oral Polio Vaccine in Panama

**DOI:** 10.3390/v13071355

**Published:** 2021-07-13

**Authors:** Magda Rojas-Bonilla, Angela Coulliette-Salmond, Hanen Belgasmi, Kimberly Wong, Leanna Sayyad, Everardo Vega, Fabian Grimoldi, M. Steven Oberste, Ricardo Rüttimann

**Affiliations:** 1 Hospital de Especialidades Pediátricas, Servicio de Infectología Pediátrica, Panama City, Panama; magda3377@gmail.com; 2 Centers for Disease Control and Prevention, Division of Viral Diseases, Atlanta, GA 30329, USA; ftt6@cdc.gov (E.V.); mbo2@cdc.org (M.S.O.); 3 United States Public Health Service, Rockville, MD 20852, USA; 4 IHRC, Inc., Atlanta, GA 30303, USA; Contracting Agency to the Division of Viral Diseases, Centers for Diseases Control and Prevention, Atlanta, GA 30329, USA; ywp8@cdc.gov (H.B.); nvj5@cdc.gov (K.W.); 5 Cherokee Nation Assurance, Tulsa, OK 74116, USA; Contracting Agency to the Division of Viral Diseases, Centers for Diseases Control, and Prevention, Atlanta, GA 30329, USA; pqd0@cdc.gov; 6 DVM, Quality Assurance Manager, VacciNet, Panama City, Panama; fabian.grimoldi@vaccinet.org; 7 Fighting Infectious Diseases in Emerging Countries, Miami, FL 33145, USA; rruttimann@fidec-online.org

**Keywords:** polio, poliovirus, novel OPV2, environmental surveillance

## Abstract

Environmental surveillance was recommended for risk mitigation in a novel oral polio vaccine-2 (nOPV2) clinical trial (M5-ABMG) to monitor excretion, potential circulation, and loss of attenuation of the two nOPV2 candidates. The nOPV2 candidates were developed to address the risk of poliovirus (PV) type 2 circulating vaccine-derived poliovirus (cVDPV) as part of the global eradication strategy. Between November 2018 and January 2020, an environmental surveillance study for the clinical trial was conducted in parallel to the M5-ABMG clinical trial at five locations in Panama. The collection sites were located upstream from local treatment plant inlets, to capture the excreta from trial participants and their community. Laboratory analyses of 49 environmental samples were conducted using the two-phase separation method. Novel OPV2 strains were not detected in sewage samples collected during the study period. However, six samples were positive for Sabin-like type 3 PV, two samples were positive for Sabin-like type 1 PV, and non-polio enteroviruses NPEVs were detected in 27 samples. One of the nOPV2 candidates has been granted Emergency Use Listing by the World Health Organization and initial use started in March 2021. This environmental surveillance study provided valuable risk mitigation information to support the Emergency Use Listing application.

## 1. Introduction

Vaccination campaigns with oral polio vaccines (OPVs) have been a part of the global polio eradication initiative by the World Health Organization (WHO) since 1959. Specifically, in 1974, the WHO formulated an Expanded Programme on Immunization to guide programs in developing countries and improve vaccination coverage. As a result of those programs, in 1990 approximately 80% of 1-year-old children had received three doses of OPV and the global morbidity and mortality associated with poliomyelitis decreased considerably [1]. In Panama the prevalence of polio is zero and the country is free from the disease. The last case of wild poliovirus type 2 in Panama was reported in 1971 and in 1972 there was a report of a non-determined wild poliovirus [2].

However, momentum toward poliovirus (PV) global eradication has been impacted by increasing paralytic poliomyelitis cases due to circulating vaccine-derived polioviruses (cVDPV), with 1088 cVDPV cases reported in 2020 compared to 378 and 105 cases in 2019 and 2018, respectively [3]. This increase followed global withdrawal of the Sabin 2 strain from the trivalent oral polio vaccine (OPV) in 2016, as the type 2 vaccine component is more frequently associated with VDPV emergence and circulation. The international spread of polio continues to be designated as a Public Health Emergency of International Concern by the WHO and specifically, cVDPV2 outbreaks are a major concern [4]. The on-going COVID-19 pandemic further complicates PV global eradication efforts due to indirect effects on vaccine supply, financial support, and immunization activities [5]. An additional strategy, environmental surveillance, has been applied by the Global Polio Eradication Initiative for decades to complement the acute flaccid paralysis surveillance with enhanced sensitivity in detecting PVs in the absence of acute flaccid paralysis cases [6]. Environmental surveillance can also assist in identifying residual wild type PV transmission, including excretion from individuals not showing clinical signs of paralysis and in the detection of VDPVs [7].

To mitigate emergence of new VDPV2s, a Consortium for Novel OPV developed candidate OPV strains that are genetically more stable than the Sabin strains and, thus, less likely to revert to neurovirulent phenotype [8]. The nOPV2 vaccine candidates were developed by a consortium of scientists from different institutions such as the UK National Institute for Biological Standards and Control (NIBSC), the US Centers for Disease Control and Prevention (CDC) and the University of California, San Francisco (UCSF), and Bio Farma. Clinical trials were held in Belgium and Panama, funded by the Bill & Melinda Gates Foundation [9]. Phase 1 study took place from May to August 2017 at a container park named “Poliopolis” at the University of Antwerp, this was the first-in-human Phase I, blinded, single-center trial. Its objective was to evaluate the safety and the immunogenicity of two nOPV2 candidates in healthy adult volunteers. The two nOPV2 candidates were live-attenuated serotype-2 polioviruses derived from a modified Sabin type-2 infectious cDNA clone propagated in Vero cells, they had numerous modifications aimed at improving the stability of the vaccine candidate by inhibiting the recombination and reducing replicative fitness, also aimed at reducing transmission. A total of 30 subjects voluntarily spent almost a month living in a 20-bed container village specially conditioned to control the environmental and health risks posed by the study of the novel vaccines, subjects were divided in two groups of fifteen participants who had received IPV as children. Subjects stayed in Poliopolis up to 28 days, or until no poliovirus was detected in any of their stool samples. The laboratory evaluated the induction of protective antibodies and analyzed the shedding of the virus in stool. The study is a step forward in the development of new OPVs in more than fifty years [10].

Given that the aforementioned trial included adults only, a phase 2 study (ID number: M5-ABMG) to evaluate the safety and immunogenicity of two novel OPV2 (nOVP2) candidates compared to a monovalent Sabin OPV was carried out in infants and children in two centers in Panama. Between 19 September 2018 and 30 September 2019, investigators carried out a single-center, multi-site, partly masked, randomized clinical trial in two groups of healthy children (1 to 4-year-old and 18 to 22-week-old infants) to evaluate the safety of the nOPV2 vaccine candidates in infants and young children after administering one or two doses of each dosage level (high or low) and to compare the results with a control sample comprising children with similar age who had received one or two doses of monovalent Sabin (mOPV2) in a prior study (informally called M2), which had taken place between 23 October 2015 and 29 April 2016, and was designed to be used as a historical control for the M5-ABMG study. Another objective of the M5-ABMG study was to evaluate the immunogenicity of a single dose of each of the two nOPV2 vaccine candidates in infants (18–22 weeks of age) after having been previously vaccinated with 3 doses of bOPV and 1 dose of IPV, and to compare these results with the mentioned cohort that served as a control sample. All subjects received one OPV2 vaccination and subsets received two doses 28 days apart. At days 0, 7, 28, and 56, type 2 poliovirus neutralizing antibodies were measured, and stool viral shedding was assessed up to 28 days post-vaccination [11].

The added value from the findings to the underlying reference populations is that participants who had been vaccinated with polio vaccines and were given a new dose of nOPV2 may have a boost for their immunity to poliovirus type 2. Regarding public health, these studies had contributed to the knowledge related to immunogenicity of recently developed OPV2s, supporting that they should be studied further, since they have proven to be viable, effective, and safe, and have the potential to be used in the event of a type 2 circulating vaccine-derived poliovirus outbreak.

Environmental surveillance for risk assessment, as an additional safety measure in clinical studies based on population dynamics and in the context of the trial settings, was recommended by the WHO Containment Advisory Group (CAG). The CAG concluded that environmental monitoring for PV should be implemented to check the duration and amount of shedding (using non-polio enteroviruses [NPEVs] as a proxy indicator for environmental surveillance sensitivity) around the nOPV2 phase 2 clinical trial sites in countries performing these trials [12]. Environmental surveillance applied as risk mitigation for the M5-ABMG Clinical Trial, referred to as “ES-M5”, was established to assess shedding of nOPV2 into community sewage and to understand the potential risks to the community following the vaccination of children and infants with the nOVP2 candidates during the M5-ABMG phase 2 clinical trial in Panama. This document focuses only on the environmental surveillance effort.

## 2. Materials and Methods

### 2.1. Study Design: Environmental Surveillance Sites and Participants

To monitor excretion and potential circulation of Sabin-like strains in the sewage during the M5-ABMG clinical trial, the ES-M5 risk assessment using environmental surveillance was conducted from November 2018 to January 2020 in five sites in Panama [the name of each city or township in parentheses]: Las Mendozas (La Chorrera), Villa Real (La Chorrera), David (Chiriquí), Las Lomas (Chiriquí), Nuevo Tocumen (Panama City). Ethically, to protect the possible identification of the study participants, maps are not shown.

The participants of the study were 5- to 8-week-old infants who were given three doses of bOPV and one dose of IPV at the age of 18 to 22 weeks; and then, were given the candidate vaccine of the study. Moreover, 1- to 4-year-old children who have already completed the routine polio vaccination scheme of the country, then received two doses of the candidate vaccine of the study.

The sewage system in much of Panama consists of small, local treatment plants, rather than large central facilities. The goal was to capture wastewater effluent from as many trial participants as possible in one location before local effluent treatment. Environmental surveillance collection sites were chosen after establishing the location of areas where most of the M5-ABMG trial subjects receiving the nOPV2 vaccines resided. The environmental sites were chosen in relation to the residential areas of the participants receiving nOPV2 vaccines with considerations regarding the location of the households where children were living and the location of the collection point before the local treatment plant. These are densely populated, low- income areas. Field visits were performed to identify sewer networks that aligned to the requirements needed for sampling, a modified approach with respect to the WHO Global Polio Laboratory Network (GPLN) guidelines for environmental surveillance [13]. Details of the collection sites and number of samples from a total of 997 participants are shown in Table 1.

### 2.2. Collection Method and Frequency

Local personnel were trained regarding safe and proper sample collection, handling, and shipping conditions. Two 1-L sewage samples for each site were collected using the grab sampling method at the inlet of local treatment plants where the highest number of study participants were co-located, as per the guidelines for environmental surveillance for the detection of polio in the presence of a sewer network [13]. Samples were stored on ice packs inside a cooler for transport. Monthly sampling for one year, the minimum recommended [13], using the grab sampling method was determined to be a feasible approach to adequately support the clinical trial versus other methods (i.e., composite). All samples were sealed and transported at 2–8 °C from the field to the laboratory supporting the clinical trial (Cevaxin), located in Panama City, where samples were frozen at ≤−20 °C until shipment on dry ice to the polio laboratory at the U.S. Centers for Disease Control and Prevention (CDC-Atlanta), within two to three weeks of collection.

Baseline samples were collected in Panama and La Chorrera sites before the start of the M5-ABMG clinical trial in November 2018 (n = 3). Monthly collections during the M5-AMBG trial vaccination took place during February to August 2019 (n = 25). Collections continued for five months after the M5-ABMG trial ended, during September 2019 to January 2020, to detect viral shedding (n = 21). All samples were collected before 9:00 a.m. to provide control in this parameter, in addition to the morning hours being an accepted approach to collect consolidated human waste using the grab sample method. Samples were not collected in December 2018, January 2019, and April 2019 due to holidays and vacations of the personnel; however, samples were collected as required within the 30 to 45 days period following vaccination. For each date and site shown in Table 2, individual samples were processed for a total n = 49. Two, 1-L samples were taken for each site on the dates shown in Table 2, mixed, and assayed. Two, 1 L sewage samples were collected to assure having enough sample for processing and a back-up was frozen.

### 2.3. Laboratory Analyses

Laboratory analyses of the environmental samples were conducted at the CDC using the standard WHO environmental surveillance methods [14]. Prior to processing, the two 1-L sewage samples were thawed at room temperature (approximately 25 °C) and mixed for at least 15 min in a sterile beaker on a stir plate using a sterile stir bar. Once thoroughly mixed, 500 mL of the sample was processed using the two-phase separation [14], and antibiotics were added to the concentrate at final concentrations of 100 IU/mL penicillin, 100 µg/mL streptomycin, and 50 µg/mL gentamycin. The remaining sample was frozen at −20 °C as back-up for any repeated processing. A portion of the resulting concentrates (500 µL) were inoculated into cell culture on the same day for enterovirus isolation. PVs and NPEV were isolated according to the recommended WHO poliovirus isolation protocol using L20B cells (recombinant murine cells that express human poliovirus receptor) and human rhabdomyosarcoma cells (RD), followed by detection and intra-atypic differentiation of polioviruses by real-time RT-PCR [15]. The ITD assay and algorithm will identify nOPV2 as a PV2, of which the sample would then be sequenced for confirmation.

### 2.4. Results Reporting

Results of the ES-M5 (code: 827) study were reported to the sponsor, regulatory authorities of Panama, the Ministry of Health of Panama with the approval of the Ministry of Health on 19 December 2018), and local ethics committee, along with an explanation of the results.

## 3. Results

### PV and NPEV Isolation

**Figure 1:**
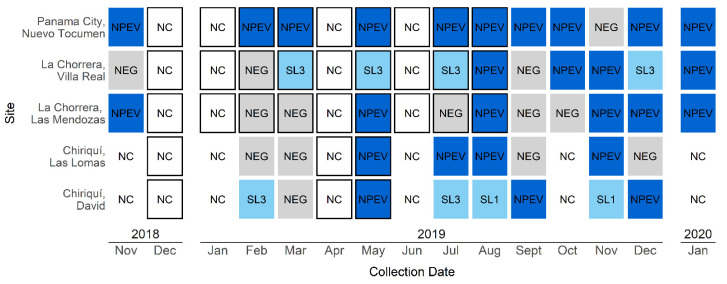
Detection of poliovirus and non-polio enteroviruses from sewage samples, by month and by site. Black outline indicates *immunization for M5 ABGM trial*. NC, no collection; NPEV, non-polio enterovirus; NEG, negative; SL3, Sabin-like type 3 poliovirus; SL1, Sabin-like type 1 poliovirus.

In total, 49 wastewater samples were collected. Sabin-like type 1 poliovirus strains, defined by WHO as “any poliovirus isolate from human or environmental sample with any nucleotide difference from Sabin less than the number that meets the definition of a VDPV” [16] were detected in two samples and Sabin-like type 3 strains were detected in six samples (Figure 1). No Sabin-like type 2 strains were detected in the samples from any of the sites.

Panama City collection site: No PVs were detected during the environmental surveillance period at the Nuevo Tocumen site. NPEV were detected in 91% (10 of 11) of the samples.

La Chorrera collection sites: No PVs were detected during the environmental surveillance period at the Las Mendozas and Villa Real sites. At the Las Mendozas site, NPEVs were detected in 55% (6 of 11) of the samples. At the Villa Real site, Sabin-like PV was detected in March, May, July, and December 2019. Real-time PCR on the viral isolates identified all of them as Sabin-like type 3 PV. Either NPEVs alone, or NPEVs and Sabin-like viruses, were detected in 73% (8 of 11) of the samples from Villa Real.

Chiriqui collection sites: No PVs were detected during the environmental surveillance period at the Las Lomas site. The samples from the David site tested positive for a Sabin-like strain in samples collected in February 2019 and in one sample collected in July 2019. Real-time RT-PCR identified the presence of Sabin-like type 3 PV in the February 2019 and July 2019 samples. Additionally, one sewage sample collected in August 2019 and another from November 2019 tested positive for Sabin-like type 1 PV. NPEV and/or Sabin-like viruses were detected in 50% (4 of 8) of the samples collected at Las Lomas and in 88% (7 of 8) of samples from David.

## 4. Discussion

This is the first example of PV environmental surveillance in Central America and specifically, in the context of a clinical trial. No Sabin-like type 2 strains were detected in sewage samples during the trial period, suggesting relatively low risk of transmission of nOPV2-related viruses from study subjects to the community. The ES-M5 study was a risk assessment mitigation measure in the evaluation of the nOPV2 vaccine candidates administered as part of the M5-ABMG clinical trial in response to the unique context created by the confluence of wild type 2 PV eradication certification, OPV2 withdrawal, and WHO PV containment guidelines [8].

Sabin-like PVs were detected in sewage samples, which was expected because bivalent OPV is used as routine immunization in Panama at 18 months and at four years of age, as an adjunct to hexavalent vaccine containing IPV administered at two, four, and six months of age. Moreover, on 21st to 26th October of 2019 there was a campaign to improve polio vaccination coverage, and in Panama Oeste (West) 2075 doses were administered in children of ages 0 to 5 years and 10 years-old, including oral polio vaccine, IPV and hexavalent [17]. Sabin-related PVs are expected in environmental surveillance samples from populations vaccinated with OPV [18]. The presence of NPEVs in sewage waters was also expected due to the virus’s presence in human feces. NPEV detection has been used as an indicator of the appropriateness of an environmental surveillance sampling site with an acceptably sensitive site yielding an enterovirus in ≥50% of samples over a 6-month period [19]. Thus, sensitivity of ES-M5 was demonstrated by detection of NPEV and Sabin-like PVs. On the other hand, lack of detection of nOPV2, in a context of a sensitive environmental surveillance system, suggests a low risk for community transmission up to 3.5 years after cessation of OPV2 use. NPEV detection is indicative of positive (method worked correctly and site selection was appropriate). A deeper analysis to relate the lack of poliomyelitis and our ES findings is beyond the scope of this paper and would require a larger sample size. The purpose of this paper was to demonstrate environmental surveillance acting as a “safety net” for the clinical trial.

One substantial limitation of this study is that many of the study participants were observed to be in diapers; therefore, their feces would not reach public sewage. A survey of the number of trial participants using diapers was not collected due to privacy reasons and as a consequence, the staff was not allowed to enter the homes of the children wearing diapers. Another limitation of the study was that the sample was small, and no biostatistician was involved in the process. An additional challenge was that the established sewer network and local treatment plants prevented an untreated sewage sample from being collected as a representative sample from a larger population in which the study participants lived. However, the localized sewer system allowed us to specifically target sewage from a large number of clinical trial participants.

Published data from two recent studies in young children and infants (phase 4 study with monovalent Sabin OPV2, and a phase 2 study with low and high doses of two nOPV2 candidates) indicate that the nOPV2 candidates produce lower stool shedding rates 28 days after vaccination than those observed with monovalent OPV2 [11]. The findings of the ES-M5 study are consistent with these findings since no nOPV2 strains were detected in any of the months that encompassed the ES-M5 study. The PVs identified in sewage corresponded only to Sabin-like PV types 1 and 3 used at that time in Panama in their childhood vaccination scheme.

Since there are considerable limitations to our study, it is not possible to rule out that some nOPV2 strains might have circulated among the population after receiving the nOPV2. A more extended use of nOPV2 in future vaccination campaigns would provide more information, necessary to assess the value of this novel vaccine and its potential role in the global polio eradication strategy in the near future.

## 5. Conclusions

The risk mitigation information provided by the ES-M5 added a community safety component to the existing nOPV2 safety data. Additionally, sensitivity was demonstrated by detection of NPEV and Sabin-like polioviruses. As we get closer to the goal of global eradication and certification, the continuation of high-quality environmental surveillance at existing sampling sites and the expansion of environmental surveillance in the final reservoirs may increase the sensitivity of overall PV detection [20] and help ensure the eradication of polio worldwide. Adding to the eradication efforts, nOPV2 vaccine has been granted a WHO Emergency Use Listing and its use in mass campaigns to control cVDPV2 outbreaks began in March 2021 [21]. Meanwhile, further nOPV2 clinical trials are underway to measure safety and immunogenicity in different populations.

## Figures and Tables

**Table 1:** Collection site characteristics and number of samples.

	Total Population ^1^	M5 Trial Participants ^2^	Number of Samples (n)
Nuevo Tocumen (Panama City)	115,151	539 subjects	11
Las Mendozas (La Chorrera)	165,000	334 subjects	11
Villa Real (La Chorrera)			11
David (Chiriquí)	144,858	124 subjects	8
Las Lomas (Chiriquí)			8
Total	425,009	997	49

^1^ Population surrounding the study participants but after the sewage is released from the local treatment plant. ^2^ The number of clinical trial participants within the community where the sewage was collected upstream from local treatment plants.

**Table 2:** Timing for vaccination and sample collection.

SITES	Month	Nov 2018	Dec 2018	Jan 2019	Feb 2019	Mar 2019	Apr 2019	May 2019	Jun 2019	Jul 2019	Aug 2019	Sep 2019	Oct 2019	Nov 2019	Dec 2019	Jan 2020
**Panama City**	**Vaccination**	**-**	**X**	**X**	**X**	**X**	**X**	**X**	**X**	**X**	**X**	**-**	**-**	**-**	**-**	**-**
	**ES sample**	▲	**-**	**-**	▲	▲	**-**	▲	**-**	▲	▲	▲	▲	▲	▲	▲
**La Chorrera**	**Vaccination**	**-**	**X**	**X**	**X**	**X**	**X**	**X**	**X**	**X**	**X**	**-**	**-**	**-**	**-**	**-**
	**ES sample**	∆	**-**	**-**	∆	∆	**-**	∆	**-**	∆	∆	∆	∆	∆	∆	∆
**David**	**Vaccination**	**-**	**X**	**-**	**-**	**-**	**X**	**X**	**-**	**-**	**-**	**-**	**-**	**-**	**-**	**-**
	**ES sample**	**-**	**-**	**-**	∆	∆	**-**	∆	**-**	∆	∆	∆	**-**	∆	∆	**-**

**X** = immunization for M5 ABGM trial; ∆ = sample collection (two sites); ▲ = sample collection (one site); **-** = no collection.
